# Transactions of the Medical Society of the State of New York, during Its Annual Session, Held at Albany, February 5, 1850

**Published:** 1850-06

**Authors:** 


*Transactions of the Medical Society of the State of New York, during its Annual Session, held at Albany, February* 5, 1850. 8vo. pp. 276.

This volume embraces, first, several reports and voluntary communications made to the Society, and referred to the publishing committee; and, second, the proceedings at the annual session, in February 1850. Of the former we will proceed to give an enumeration, with a few comments. We have neither leisure, nor space for analyses or extended criticisms. First in order is the address by the late president of the Society, Alexander H. Stevens, M.D. The subject selected for the occasion was the *Public Health*—a subject peculiarly appropriate, inasmuch as the address was delivered at the capitol, in the Hall of the Assembly, a large portion of the members of the Legislature being present. Appended to the address are some remarks on "the sanitary construction of country dwellings, ventilation, drainage, etc." The address and the appendix contain some valuable suggestions with which it were desirable that the public should be familiar. In treating of public health the author confines his view to the *financial* aspect of the subject, presenting considerations to show how, and in how far, the wealth of the State is affected by ignorance and neglect of the laws regulating the physical welfare of its population.

Art. II. *On the Communicability of Asiatic Cholera,* by ALEXANDER H. STEVENS, M.D.

With due deference to the learned author, we must say that, in our opinion, this is a weak defence of the doctrine of the contagiousness of cholera; but the subject appears to us hardly to admit of very powerful argumentation. We designed, when we first read this article, to review it at length, and discuss fully the subject of which it treats; but we must defer carrying out this intention for some other occasion. We will not, however, forego briefly noticing the manner in which Prof. Stevens states the logical position of the doctrine of non-contagion. We quote the two first paragraphs of the paper as follows:

"Two theories, and two only have been conceived to account for the existence of the Asiatic cholera. It is the design of the following paper to examine each of these in detail, and to endeavor to decide to which of them truth belongs.

"According to the first theory, the disease is caused by a peculiar state of the atmosphere extending over vast regions, imperceptible to the senses, and giving no evidence of its existence by statical, chemical or medical tests, or indeed in any other way than in the causation of the cholera. The starting point of the doctrine is therefore a double hypothesis. It assumes without proof: 1st. That this peculiar state of the atmosphere exists. 2d. That it is the cause of cholera. These propositions have no other support than that which they derive from each other. Cholera exists, therefore there is a peculiar state of the atmosphere. There is a peculiar state of the atmosphere, therefore cholera exists. This is palpably only reasoning in a circle."

Now, in the first place, it is far from being tne case that "two theories only have been conceived to account for the existence of the Asiatic cholera." To attribute it to a peculiar state of the atmosphere, is not the only alternative, provided the doctrine of contagion be repudiated. Various hypotheses have been suggested which do not involve states of the atmosphere. But without enlarging on this point, what non-contagionist ever reasoned in the way he is represented as reasoning in the foregoing extract? Who ever assumed that a peculiar state of the atmosphere exist, and, ergo, cholera exists; and who ever argued from the mere existence of cholera that there is a peculiar state of the atmosphere? We might say that this statement is unfair, but it is too nonsensical to complain of on that score. The question is simply this, do observations and reasoning sustain, or do they disprove the supposition that cholera is diffused by contagion? This question, in the present state of knowledge, has nothing to do with inquiries as to the most probable theory of causation, provided the communicability of the disease be disproved. To render the attitude of those who disbelieve in the contagiousness of cholera ridiculous, a more ingenious artifice is requisite than is exhibited in the passages we have quoted.

III. *An Historical Sketch of the State of Medicine in the American Colonies, from their first settlement to the period of the Revolution.* By John B. Beck, M.D., etc.

This is an interesting and valuable contribution. We could wish it were more full. It is gratifying to see that attention is being directed to the subject of American medical history. Prof. N. S. Davis, of Chicago, Ill., is engaged in preparing a work on this subject, which is to appear in successive numbers of the Northwestern Medical and Surgical Journal. He is fully competent to the task, and will render an useful service if he perseveres to the completion of the undertaking.

IV. *Contributions to the vital Statistics of the State of New York.* By Lemuel Shattuck, Esq., of Boston, Mass.

Mr. Shattuck (whose name is identified with the subject of vital Statistics) presents in this paper a series of tables, exhibiting (*a*) The number of inhabitants in the several counties, enumerated in the National censuses of 1820, 1830, and 1840; and in the State censuses of 1825,

1835 and 1845. (*b*) The area of each county in square miles and square acres; and the number of inhabitants to the square mile in 1825, 1835, and 1845. (*c*) The number of births in the several counties returned in the State censuses of 1825, 1835 and 1845; and their relation to the population. (*d*) The number of marriages in the several counties, returned in the censuses of 1835 and 1845; and their relation to the population. (*e*) The number of deaths in the several counties, returned in the censuses of 1825, 1835 and 1845; and their relation to the population. (*f*) The population of the several cities in the State in 1820, 1830 and 1840; and in 1825, 1835 and 1845. (*g*) The number of births, marriages and deaths, returned in the State censuses of 1825, 1835 and 1845; and their relation to the population in the several cities of the State.

Mr. Shattuck, from the investigation of the censuses upon which these tables are based, arrives at the following conclusions respecting the statisical data attained in that way:

"The result of this examination has led to the conclusion that these census returns are not reliable for accuracy. The design for obtaining them was excellent, but it was not carried out in truthfulness; and the last attempt was the greatest failure. The same results have followed similar attempts elsewhere.

"My own knowledge in these matters, strengthened by this examination, has led me to conclude that full and accurate returns of this kind cannot be obtained by personal inquiry, as these were obtained, after the expiration of a year. Owing to the locomotive habits of our people, causing frequent removals from one place to another, and from other causes, many births, marriages and deaths, will escape notice, and render the returns defective. The only effectual mode of preventing errors in matters of this kind is such a plan of public registration as will secure the record of every birth, every marriage, and every death, with all the important facts in relation to each, *at or near the time in which they occur*. Such a system of registration should be the foundation of all sanitary laws. Without it no accurate information concerning the population, or its sanitary condition can be ascertained.

"Some persons have desired that an attempt should be made, in the census of the United States for this year, to obtain the number of deaths. The results here presented will show the probable value of the information which might be obtained in this way. I have doubted the expediency of such a measure for two reasons; one because the information when obtained could not be relied upon for accuracy; and another because, if it could be accurately obtained for the year 1849, (that being a very sickly year) it would give an unfair exhibition of the average sanitary condition of the country. A strong desire was, however, expressed by the Census board of Washington, that measures should be provided for obtaining the information; and a separate schedule was prepared, at their request, for the purpose."

V. *Annual Address, delivered before the Albany County Medical Society, November*, 1749. By James McNaughton, M.D., President.

The subject of this address is *Epidemic Cholera*. It is mainly a report of the disease as it prevailed at Albany. The topographical situation of cases as they successively occurred, are given, in order to determine what bearing these *facts* have upon the question of the diffusion of cholera by contagion. Prof. M. regards the evidence as conclusive against the opinion that the extension of the disease is dependent upon contagion; but he is not fully satisfied that it is not sometimes so communicated. He gives some instances of *apparent* contagion, or in which the disease seemed to be carried by some *portable*, special, morbid agent. Such instances, it will not be denied, generally occur, whenever, or wherever the epidemic prevails.

The whole number of cases occurring at Albany in 1849, which were reported to the Boaid of Health, was 8318—number of deaths, 334. Prof. M. states that in many cases of cholera which fell under his own observation, the diarrhœa was preceded by a *moist white tongue*, which he had observed as a striking characteristic of the disease in 1832. He deems this symptom important "as a means of knowing when the epidemic influence threatened the system with an attack, even earlier than the premonitory diarrhœa gives warning."

VI. *Report of the Standing Committee of the Society on Hygiene, or medical statistics*. By Charles A. Lee, M. D., Chairman.

Prof. Lee states that no assistance having been rendered him by those associated on the committee, and but few replies having been received to queries addressed to members of the profession in different parts of the State, he has come to the conclusion that the only method of collecting a body of facts which will serve any useful end, is that adopted by the Commonwealth of Massachusetts, viz. the sanitory survey of the state, performed by Commissioners appointed and remunerated by the state. He therefore confines himself to a statement of some of the considerations showing the great need of such a survey. These considerations are very forcible, and should be appreciated more fully than they are, by the medcal profession, as well as by the public. To both, the attention of the brief report of Dr. Lee may be commended. He presents a striking contrast between the provisions for the sick poor in Great Britain, and in some portions of this country. The instance related for illustration, from the latter is, (we are sorry to say) the Erie County Poor-house, as it was not long ago. We trust there is some improvement since. In conclusion,

Prof. LEE submitted the manuscript work by the late Dr. FORRY, of New-York, (committed to his hands by the talented author shortly before his decease,) in the hope that the Society might secure its publication by incorporating it in their transactions. The Committee on Publication, as we are informed, were desirous of so doing, but it was found that the size of the volume of Transactions would be thereby increased, so as to jeopardize its being issued at the expense of the State. It is to be hoped that the work will appear in the next volume of the Transations of the American Medical Association, a vote to that effect having passed, with the proviso that the pecuniary resources of the Association are adequate to that eud.

VII. *On the Vital Statistics of the city of Brooklyn.* By CHAS. S. J. GOODRICH. M. D.

The whole number of deaths from cholera in 1849, was 650 ; 500 adults, 150 children ; 325 males, and 325 females. Of the adults 75 were natives of the United States, 391 from Ireland and Germany, and 34 from other countries.

VIII. *Medical Topography of the County of Montgomery.* By JOSEPH WHITE, M. D.

Dr. WHITE states that the inhabitants of Montgomery County, as a whole, are less afflicted with *consumption* than the adjoining counties, which are more elevated ; but one village in the County presents a striking exception to this remark. We quote his description of the place re ferred to, as follows :—

" There is however one exception to the exemption or less frequency of consumption in Montgomery county. In the south-west part of the county, about three miles north of Sharon Springs, is the village of Ames, containing about 250 inhabitants. It is situated upon the edge of a flat, which at this place is about one mile in breadth and formed by the junction of Sulphur Creek, which has its origin at Sharon Springs, with Canajoharie creek. These flats are somewhat swampy, (there would be no serious difficulty in draining them,) and are quite subject to fogs. There is a mill-dam across Canajoharie creek a mile and a half below the village, which sets the water back about one mile, yet it flows but little more than the natural channel of the stream. There are also upon Sulphur creek, within one mile of the village, three small dams, which flow from one to three acres of ground. The size of these ponds would seem to be too small to influence very materially the atmosphere of their vicinity. All the wells and springs in the village are slightly sulphurous, and highly charged with lime in solution, being supplied, probably by the Sulphur creek above mentioned, which runs through the village.

It is asserted by the inhabitants, and I have no doubt truly, that at least one half of all who die in that village, die of consumption, and yet, one mile, or a mile and a half from the village, the inhabitants are as free from consumption as in any section of the county. The residents of the place are mostly farmers of English descent, whose habits are like others of the same occupation, frugal and industrious, residing in dwellings as comfortable as farmers in good circumstances usually have.

IX. *Analysis of the Byron Acid Spring, near Batavia.* By Dr. George Hand Smith, of Rochester.

This spring contains free sulphuric acid in a large proportion, 59,397 of 100,000, and 32,022 parts of proto sulphate of iron and alumina. Its medical powers are stated to be highly tonic, refrigerent and astringent.

Several springs analagous in the composition of their waters, are found in this neighborhood.

X. *Report of a case of premature labor, artificially induced, etc.* By J. W. Blatchford, M. D.

(See Eclectic department of this No. of this Journal.)

XI. *Semi-annual address on Erysipelas, delivered before the Albany Co. Medical Society, in June,* 1849. By John Swinburne, M. D., Vice President.

The subject of this paper is to establish the identity of Epidemic Erysipelas, diffuse cellular inflammation, puerperal fever, and phlebitis. The author thinks that the specific characteristics of each depend upon the texture affected, the essential morbid condition being the same. He advocates the contagiousness of puerperal fever. He relies, mainly, for the support of these views, on facts cited from various authors.

XII.—*Case of Poisoning by Corrosive Sublimate.* By Benj. W. McCreedy, M. D., of New York.

This is an interesting case, which we reserve for the Eclectic department of a future No. of this Journal.

XIII.—*Notices of the Cholera at Newark, in* 1832. By John S. Dorey, M. D., in a letter to Alexander H. Stevens, M. D.

The statements contained in this paper are presented as tending to show the contagious ness of Cholera. The author gives his recollection of events that transpired eight years previous. With a strong bias in favor of the doctrine of contagion, and with a convenient looseness of expression, it may still be questioned if the narration, on the whole, does not go to disprove the communicability of the disease.

XIV.—*Notices of the Cholera at Rockaway in* 1849. By JULIUS AUERBACK, M. D., in a letter to ALEXANDER H. STEVENS, M. D.

This short paper, as well as the foregoing, was communicated by Dr. Stevens, as containing *facts* in support of the contagiousness of Cholera. The only events which appear to bear on the question of contagion, are, that the first case in Rockaway occured in the person of a Captain Hawser, captain of a small vessel plying between New York and Rockaway, that he was attacked the day after his arrival from the city, and that he had in his vessel household furniture and beds belonging to a man "*whose wife had died shortly before in New York, and was brought to this place for interment.*" (This quotation is italicised in the work.) Several cases subsequently occurred, but the report gives neither respective dates, distances of situation, nor any adequate evidence of the intercourse of the patients affected.

We cannot refrain from expressing surprise that such a paper should be seriously submitted as containing any authenticated facts relative to the question of the contagiousness of Cholera. It seems to us better calculated to bring ridicule on the affirmative of that question.

In so far as the passage italicised is concerned, the facts contained in it are opposed to the hypothesis of contagion. A captain of a vessel direct from a choleraic atmosphere, is attacked with cholera. Some household goods of a person whose wife had lately died with the disease, were on board. The body of the deceased lady had shortly before been brought from the city to Rockaway for interment, but no cholera followed. The captain of the vessel alone was attacked, while the crew, who, it is reasonable to suppose, had been brought into closer proximity with the suspected *fomites,* all escaped. The son of the captain is attacked simultaneously with the father, but, as it is not stated that he was on board the vessel, it may be presumed he was not, although if he were, it would not affect the question. Finally, both patients recover, so that there is room for incredulity as respects the diagnosis. We are sure that only a contagionist whose preconceptions prevented him from seeing aught else than what appeared to favor his peculiar bias of mind, could discern in this summary of events, anything deserving to be dignified with the name of evidence.

XV.—*On the Morbid condition of the Generative Organs, being a paper read before the Chenango Co. Medical Society.* By WM. D PURPLE, M. D., of Greene, Chenango Co., N. Y.

This communication contains the history of cases which, we agree with the writer in thinking, are too much neglected and overlooked by the medical profession. We have long been satisfied that the abuse of the sexual function, and especially its unnatural excitation, is the true source of not a few of the maladies which physicians are called upon to treat in young persons of both sexes, and which are not thus referred. We are fully senslble of the delicacy and difficulty of tracing disorders to this source in individual cases, and hence it is that the attempt is not made, or, if made, frequently proves unsuccessful. The physician, to discharge his duty in this particular, must not be restrained by false modesty, the fear of giving offence, or of wounding the feelings of patients or friends by an unfounded imputation ; nor must he remain satisfied with a tame, superficial enquiry. He must question directly and plainly, cross-question, and persist until he believes he has arrived at the truth. Were this course faithfully pursued, our art would accomplish, we are persuaded, much more for the moral and physical welfare of our patients than it does at present. Many affections that now tire the patience of the practitioner, and bring opprobrium on the art, would be found to be speedily remediable. This is our belief, based, we are free to confess, chiefly on the observation of cases in which our suspicions have exceeded our knowledge. It is proverbially easier to preach than to practice, and, in this instance, we are sorry to say that we cannot enforce our precepts by reference to our own professional observations.

XVI.—*Account of an Operation for the removal of an Ovarian Tumor.* By ALDEN MARCH, M. D., etc.

See Eclectic department of this number of this Journal.

XVII.—*Observations on various subjects in Forensic Medicine.* By JENKS S. SPRAGUE, M. D.

The greater portion of this paper is devoted to an account of two cases of persons found dead under circumstances giving occasion for the suspicion of their having been murdered, which, in the opinion of the writer, received inadequate investigation from the professional gentlemen in attendance. In connection with this opinion, the writer takes oceasion to indulge a fling at the medical school, at which one of the incompetent medical gentlemen particularly referred to, had lately graduated. This may be in excellent taste, but, if so, our judgment is greatly at fault. It is any thing but a good sign, in our humble estimation, to recount the faults, or deficiencies, of our neighbors. It may argue a want of charity, but we cannot but think that not a few of those who are constantly prating of the ignorance and incompetency of their professional brethren, would be better occupied in self-improvement.

Dr. Sprague advocates the appointment of a suitable number of medical examiners in every county, selected with reference to their fitness and acknowledged qualifications for medico-legal examinations. This proposition, which was first made, if we recollect aright, by Prof. Stevens, of N. Y., strikes us as a good one, and we should be glad to see it adopted by the State.

XVIII. *Brief Notices of the Medical Topography and Diseases of Washington county.* By HIRAM CORLISS, M.D.

Dr. Corliss details, briefly, several cases of epidemic erysipelas. Every case treated by him recovered. His treatment consisted of quinine, morphia, and wine.

XIX. *Notices of Deceased Members.*

This embraces a short biography of the late Peter Wendell, M.D.; a sketch of the life of the late Dr. A. Brigham, by Prof. Coventry, which has appeared in the pages of this Journal; and a notice of the decease of Dr. Joseph A. Gallup, of Woodstock, Vt., an honorary member of the State Medical College.

*Of the proceedings of the Society we shall give some account, in addition to the synopsis formerly given (No. for March,* 1850), *in our next number.*

